# Polymorphisms in Lysyl Oxidase Family Genes Are Associated With Intracranial Aneurysm Susceptibility in a Chinese Population

**DOI:** 10.3389/fendo.2021.642698

**Published:** 2021-07-28

**Authors:** Chun Luo, Chongyu Hu, Bingyang Li, Junyu Liu, Liming Hu, Rui Dong, Xin Liao, Jilin Zhou, Lu Xu, Songlin Liu, Yifeng Li, Dun Yuan, Weixi Jiang, Junxia Yan

**Affiliations:** ^1^Department of Epidemiology and Health Statistics, XiangYa School of Public Health, Central South University, Changsha, China; ^2^Department of Neurology, Hunan People’s Hospital, Changsha, China; ^3^Department of Information Statistics, Changsha Hospital of Traditional Chinese Medicine (Changsha Eight Hospital), Changsha, China; ^4^Department of Neurosurgery, XiangYa Hospital, Central South University, Changsha, China; ^5^Department of Scientific Research, The People’s Hospital of Guangxi Zhuang Autonomous Region, Nanning, China; ^6^Hunan Provincial Key Laboratory of Clinical Epidemiology, XiangYa School of Public Health, Central South University, Changsha, China

**Keywords:** intracranial aneurysm, lysyl oxidase family genes, *LOX* gene, *LOXL2*, polymorphism

## Abstract

**Purpose:**

Intracranial aneurysms (IA) comprise a multifactorial disease with unclear physiological mechanisms. The lysyl oxidase (*LOX*) family genes (*LOX*, *LOX–like 1–4*) plays important roles in extracellular matrix (ECM) reconstruction and has been investigated in terms of susceptibility to IA in a few populations. We aimed to determine whether polymorphisms in *LOX* family genes are associated with susceptibility to IA in a Chinese population.

**Methods:**

This case-control study included 384 patients with IA and 384 healthy individuals without IA (controls). We genotyped 27 single nucleotide polymorphisms (SNPs) of *LOX* family genes using the Sequenom MassARRAY^®^ platform. These SNPs were adjusted for known risk factors and then, odds ratios (OR) and 95% confidence intervals (CI) were evaluated using binary logistic regression analysis.

**Results:**

The result showed that *LOX* rs10519694 was associated with the risk of IA in recessive (OR, 3.88; 95% CI, 1.12–13.47) and additive (OR, 1.56; 95%CI, 1.05–2.34) models. Stratified analyses illustrated that *LOX* rs10519694 was associated with the risk of single IA in the recessive (OR, 3.95; 95%CI, 1.04–15.11) and additive (OR, 1.64; 95%CI, 1.04–2.56) models. The *LOXL2* rs1010156 polymorphism was associated with multiple IA in the dominant model (OR, 1.92; 95%CI, 1.02–3.62). No associations were observed between SNPs of *LOXL1*, *LOXL3*, and *LOXL4* and risk of IA.

**Conclusion:**

*LOX* and *LOXL2* polymorphisms were associated with risk of single IA and multiple IA in a Chinese population, suggesting potential roles of these genes in IA. The effects of these genes on IA require further investigation.

## Introduction

Intracranial aneurysms (IA) are cystic bulges caused by local defects or increased pressure in the intracranial artery wall (1). Unruptured intracranial aneurysms (UIA) occur in 3–5% of the global population and in 7% of Chinese persons aged 35–75 years (2–5). The annual risk of IA rupture is 0.5–1.8% (6, 7). The rupture of an IA can result in aneurysmal subarachnoid hemorrhage (aSAH), which can be fatal in 50% of patients and cause 33% of severe sequelae (8, 9). Costs associated with aSAH supervision exceed £510 million annually in the UK (10). Therefore, risk factors linked to IA should be determined as soon as possible to screen and treat IA more effectively.

In addition to the established acquired risk factors of cigarette smoking and hypertension, genetic factors also play significant roles in IA (3). Among patients with IA, 4% and 8% have first- and second-degree relatives with IA, respectively (11). The predicted probability of IA is higher among individuals with hereditary pathologies such as autosomal dominant polycystic kidney disease (ADPKD) and Marfan syndrome (12). The extracellular matrix (ECM) is an indispensable element for maintaining arterial wall stability. A disrupted balance between ECM synthesis and degradation may be the pathophysiological basis for IA formation (13, 14). For example, some single nucleotide polymorphisms (SNPs) of endothelin receptor type A (*EDNRA*), alpha 1 type III collagen (*COL3A1*), transcription factor SOX-17 (*SOX17*), and lysyl oxidase-like 2 (*LOXL2*), which are mainly involved in ECM reconstruction, are associated with IA (15–18). The *LOXL2* gene belongs to the lysyl oxidase (LOX) family that comprises *LOX* and lysyl oxidase-like 1–4 (*LOXL1*, *LOXL2*, *LOXL3*, and *LOXL4*) (19). Lysyl oxidase can initiate covalent crosslinking between soluble collagen and elastin monomers that are converted into insoluble, stable fibers in the ECM, thereby ensuring the structural and functional stability of the arterial wall (20). Dysregulated LOX activity may play significant roles in cardiovascular diseases such as atherosclerosis and aneurysms (21), which are mediated by cross-linked structural ECM proteins. Thus, the *LOX* family of genes might be associated with IA.

So far, few population studies have explored associations between polymorphisms in *LOX* family genes and IA, and the results were inconsistent. For instance, associations between *LOX* gene polymorphisms and IA have not been found in Japanese, Central European, and South Indian populations (22–24). However, a recent population study in Korea found three SNPs of *LOX* (rs2303656, rs3900446, and rs763497) were significantly associated with IA (maximum OR, 20.15; *P* = 4.8 × 10^–5^) (25). Akagawa et al. (18) examined associations between *LOXL1–4* gene polymorphisms and susceptibility to familial intracranial aneurysms (FIA) in Japan and found that rs1010156 of *LOXL2* was associated with FIA (OR, 1.49; P = 0.023). Using whole exome sequencing (WES), Wu et al. (26) also discovered that the rare variant of *LOXL2*, c.133C>T (rs142252012), is related to the FIA susceptibility in a Chinese population. Considering genetic heterogeneity among ethnic populations and that the genetic background of FIA might differ from that of sporadic intracranial aneurysms (SIA), we aimed to determine whether polymorphisms in *LOX* family genes are associated with susceptibility to IA in a Chinese population.

## Methods

### Study Population

We recruited 384 consecutive patients with IA at Xiangya Hospital of Central South University and the Hunan Provincial People’s Hospital (Chang Sha, Hunan province, China) since January 2016. The diagnosis and phenotype information of IA (number, location, and rupture status) was confirmed by magnetic resonance imaging (MRI), computed tomography (CTA), or digital subtraction (DSA) angiography. Other neurological and vascular conditions such as arteriovenous malformation and Marfan syndrome were excluded. We also randomly selected 384 healthy individuals (control), who visited a district community health service center for routine annual health check-up and had no personal or family history of IA, SAH, or other known related vascular diseases. Demographic information and clinical data were collected using a questionnaire and from medical records. None of the participants were consanguineous. Peripheral venous blood sample was collected from all participants into 5 mL EDTA–K2 anticoagulant tubes (Sanli Medical Technology Co., Ltd., Liuyang, China). All participants provided written, informed consent before the study was initiated. The study was performed under the approval of the Ethics Committee at Central South University (Permit No: CTXY–150002–1). Furthermore, to test the robust of the association results identified in this study, an additional population control was adopted (208 normal Chinese Han individuals in the 1000 Genomes Project, the genotype information was download from http://grch37.ensembl.org/Homo_sapiens/Info/Index).

### SNP Selection and Genotyping

We selected SNPs based on tag SNP and determined by Genome Variation Server 150 (http://gvs.gs.washington.edu/GVS150/index.jsp). The threshold of the LD parameter r^2^ was set at 0.8. The minor allele frequency (MAF) of all selected SNPs was > 5%. After tag SNP screening by gene name, priority was given to variants with a proven relationship to IA or SNPs located in the functional exonic region or that covered many other sites. We finally selected 27 SNPs of *LOX* family genes into this study (**Table 1**). Among them, *LOX* rs2303656, rs763497, and rs3900446 SNPs were positively related to IA in a Korean population (25), while *LOXL2* rs1010156 and rs142252012 SNPs were positive in Japanese and Chinese families, respectively (18, 26).

**Table 1:** Information of *LOX* family genes polymorphisms.

Gene (Genbank accession number)	SNPs	Position	Role	Variant		MAF^d^	Forward primer sequence (5'-3')	Reverse primer sequence (5'-3')	Length(bp)	SNPs covered by tag SNPs
				cDNA	Amino Acid					
*LOX* (NM_002317)	rs1800449(C>T)^a^	Chr5:122077513	Missense	c.473C>T	p.R158L	0.17	AGAAGTTCCTGCGCTCAGTA	TGGGCCTTTCATAAGTATCG	134	rs2288393/rs10059661
	rs2956540(G>C)^a^	Chr5:122073485	Intron	c.1035+528G>C	–	0.21	TTCACCTGTGAAACCATTCC	GAAATGGTGTCCTTCTGCTC	152	–
	rs10519694(C>T)^a^	Chr5:122071524	Intron	c.346-935C>T	–	0.05	ATGCCACATCACTCCACTTG	CTGAGGAAACTTCTCTAGAC	135	–
	rs2303656(G>T)^b^	Chr5:122070281	Intron	c.1132-113G>T	–	0.02	CTGGGCAACACAAAGAGTTC	TTTCCATAACGTCTCCAGAG	141	–
	rs763497(A>G)^b^	Chr5:122091535	Intergenic	–	–	0.13	ACATCTAGGCCTACATCGAG	TAAATGGCCCCCAACACAAG	129	–
	rs3900446(A>G)^b^	Chr5:122090980	Intergenic	–	–	0.12	AGGAAGCAAAGCTCAGGTGG	CTTGAAGTTTCCCAGTAAGG	120	–
*LOXL1* (NM_005576)	rs2165241(C>T)^a^	Chr15:73929861	Intron	c.1102+1976C>T	–	0.07	AAACTGAGCTCTCAAATGCC	CTCTCAATCAACTGGCTTCC	131	rs750460/rs4243042/ rs4886782/ rs12440667
	rs3825942(G>A)^a^	Chr15:73927241	Missense	c.458G>A	p.G153D	0.13	ACCTCCGTCTCCCAGCAAC	TAGTTCTCGTACTGGCTGAC	143	rs1078967/rs8041642/ rs8042039/ rs8041685
	rs2304721(C>A)^a^	Chr15:73948013	Intron	c.1602+111C>A	–	0.23	TGTTCATGTCCAATGTCCCC	CTGAGACCTAAATCTTCGGC	140	rs1440101/rs12594472/rs16958494
	rs12441130(T>C)^a^	Chr15:73942561	Nonsense	c.1103-293T>C	p.T446R	0.29	AGCTTACATCTCGAGCTCTG	TTCATGCTGTTTTCCCTGCC	143	rs2028386/rs4337252
*LOXL2* (NM_002318)	rs2294128(C>T)^a^	Chr8:23333413	Synonymous	c.954C>T	p.A318A	0.14	TGCCAAGTGGCCACACCTC	CATGAAGAATGTCACCTGCG	146	rs2294127/rs2294126/rs2294129/ rs3779895/rs9792317/rs10503724/ rs11775841/rs11987443/ rs17089043/ rs17089055
	rs7818494(A>G)^a^	Chr8:23366501	Intron	c.355+1496A>G	–	0.22	GTTGGAAGGGAGGATAACAG	AGAATAGCGCAGACCTCAAC	140	rs4872112/rs7835142/rs10096530/ rs10099318/rs10112621/rs11135728/ rs11992138/rs12674548/ rs12677717/ rs17089161
	rs4323477(A>G)^a^	Chr8:23324229	Intron	c.1151-1948A>G	–	0.49	ATAGACGTTCAGCCACAAGG	AGCCAACTTAAGAGCCTAGC	126	rs7829632/rs11135726/rs12676713/ rs13248926/rs13259317/rs13272473/ rs13282766/rs17088178
	rs7818416(G>A)^a^	Chr8:23373510	Intron	c.-83-5076G>A	–	0.43	CAAGAGATCCTCCTACTCAG	ACCTTTGGCAATTCATTGGC	148	rs7009281//rs11782783/rs4285498/ rs6983061/rs13252670/rs13254155
	rs1063582(G>T)^a^	Chr8:23309840	Missense	c.1708G>T	p.M570L	0.27	TCTCTTGCCTTGTTGACCAG	AGTTCTCCTCCATGGCACAC	136	rs3765215/rs3808521/ rs4273857/ rs4278162/rs4872103
	rs2280936(C>G)^a^	Chr8:23297824	3'UTR	c.219C>G	–	0.11	AGCAGCTCTGTGGACAAACC	CTACAGCTGTGTCTAAGCTC	119	rs1051157/rs7813349/rs7834641
	rs2294133(C>T)^a^	Chr8:23368232	Synonymous	c.120C>T	p.P40P	0.23	CATTACCCCGAGTACTTCCA	CATCATAGTACACCTCCACC	139	rs17089187/rs4871870/rs6557667
	rs2280935(A>C)^a^	Chr8:23297617	3'UTR	c.426A>C	–	0.37	GGAGGGTTTCATTGGAAGAG	TGACACGTGGACAAATGCGG	127	rs2280937/rs11990784
	rs1010156(T>C)^ab^	Chr8:23333428	Synonymous	c.939T>C	p.S313S	0.45	CATGAAGAATGTCACCTGCG	GTCCTCACCTCTGGCTTGTA	120	–
	rs142252012(G>A)^b^	Chr8:23368219	Missense	c.133G>A	p.H45Y	0.01	CATCATAGTACACCTCCACC	ATTACCCCGAGTACTTCCAG	138	–
*LOXL3* (NM_032603)	rs715407(T>G)^a^	Chr2:74538566	Intron	c.693-1638T>G	–	0.19	GTCCCCTTTGGAACCTTTAC	AAGCTTCCCACTTCGAGTTC	133	–
	rs6707302(C>T)^a^	Chr2:74534295	Intron	c.1939+21C>T	–	0.14	CACTATGATATCCTCACCCC	AAGGCTGTGCAATGGATACC	136	–
	rs17010021(T>A)^a^	Chr2:74534412	Missense	c.1843T>A	p.I615F	0.36	ACTCAGTGTCTTCGAGACAG	CTTCTCCCCACAGGCATTAC	120	–
	rs17010022(C>G)^a^	Chr2:74536131	Synonymous	c.1113C>G	p.L371L	0.32	ACTTCCTGATCTTTGCCATC	GTGAACAATCCTCAGCTGTG	128	–
*LOXL4* (NM_032211)	rs3793692(G>A)^a^	Chr10:98248679	3'UTR	c.242G>A	–	0.47	GGATGACTGGGTTTCCTTAC	GATGGCAAGATCACCAATCC	140	rs737656/rs737657
	rs1983864(G>T)^a^	Chr10:98257696	Missense	c.1214G>T	p.D406A	0.39	GATATGAGCGGACCCTCAG	ATGCTCAGACCCAAACTCAC	136	rs878177/rs1983866
	rs7077266(G>T)^a^	Chr10:98259282	Intron	c.702-54G>T	–	0.19	TGGCATGAAGGGCCTCTATC	GGAGTTCTTATTCGTCAGGC	151	rs3763688

SNP, Single nucleotide polymorphisms; OR, odds ratio; CI, confidence interval; MAF, Minor allele frequency −, not available.

^a^Tag SNPs; ^b^Positive variants associated with IAs or aSAH in previous studies; ^c^The second allele is the minor allele; ^d^The MAF in the HapMap-HCB population.

Genomic DNA was extracted using TIANamp Blood Genomic DNA Isolation kits (TIANGEN Biotech Co., Ltd., Beijing, China) and stored at -80°C. Primers were designed according to the SNP loci using Assign Design 3.1 software (http://agenacx.com) and their sequences are detailed in **Table 1**. Target SNPs were genotyped using the MassARRAY iPlex platform (Agena Bioscience Inc., San Diego, CA, USA). Polymerase chain reactions (PCR) were performed as described (17) in 5 μL reaction mixtures containing 1.0 μL template DNA (20–50 ng), PCR Primer mix (1 μL), 10 × PCR buffer (0.5 μL), dddH2O (1.8 μL), 25 mM MgCl2 (0.4 μL), 25 mM dNTP (0.1 μL), and 0.2 μL Taq polymerase (5 U/μL) (HotStarTaq^®^; Qiagen GmbH, Hilden, Germany). The amplification cycles were as follows: pre-denaturation for 2 min at 95°C, followed by 45 cycles of 30 sec at 9°C, 30 sec at 56°C, 60 sec at 72°C, and a final elongation step of 5 min at 72°C. The amplified products were stored at 25°C. When the PCR endpoint was reached, excess dNTPs were removed using shrimp alkaline phosphatase followed by single nucleotide extension and resin desalination steps. We identified SNP genotypes and alleles using MALDI–TOF–MS, mass spectrum peaks and MassArray TYPER 4.0 software. Finally, target genotypes were interpreted according to the peaks.

### Statistical Analysis

Data were analyzed using SPSS 23.0 (IBM Corp., Armonk, NY, USA). Continuous variables are described as means ± standard deviation (SD) and categorical variables are expressed as ratios (%). Pairs of normally distributed continuous variables were compared using Student t-tests, and distribution differences between two categorical variables were compared using chi-square or Fisher exact tests. The Hardy-Weinberg equilibrium of each SNP in the control group was assessed using chi-square tests. Pairwise LD was evaluated using the mean value of the squared correlation coefficient (r^2^) and the standardized disequilibrium coefficient (D’) in Haploview v.4.2 (https://www.broadinstitute.org/haploview/haploview) (27). A pair of SNPs was determined in strong LD under the criterion r^2^ ≥ 80%. Complete LD was determined when D’ = 1 (28). We calculated OR and 95% CI using binary logistic regression in different genetic models after adjusting for known risk factors (dominant, recessive, and additive models). The threshold of significance was set at p < 0.05. Statistical power (SP) was calculated according to the used significance level, sample size, obtained OR and the corresponding genotype carrier rate in the control group under two-sided Z test by using PASS 11 software (NCSS LLC, Kaysville, Utah, USA).

## Results

### Characteristics of Study Population

Females comprised 69.5% of the 384 patients with IA and the 384 controls and ~50% participants of both groups had hypertension (*P* > 0.05). Among the patients, 48.7% had IA located in the internal carotid arteries and 64.5% and 35.5% had single and multiple IA, respectively. The distribution of gender and hypertension did not differ significantly between patients with single or multiple IA and the controls. The mean age was lower and smoking, diabetes, and hyperlipidemia were less frequent in the patients than in the controls (*P* < 0.05). In single or multiple IA groups, approximately 50% and 20% of aneurysms were located in the internal carotid and middle cerebral arteries, respectively. **Table 2** lists the detailed characteristics of the participants.

**Table 2:** Characteristics of the study population.

Characteristics	IA			Control (n = 384)
	Single IA (n = 248)	Multiple IA (n = 136)	Total (n = 384)	
Age, years (Mean±SD)	**57.3±10.4**	**56.7±10.9**	**57.1±10.6**	66.5±2.1
Female, n (%)	166 (66.9)	101 (74.3)	267 (69.5)	267 (69.5)
Smoking, n (%)	**30 (12.1)**	**15 (11.0)**	**59 (15.4)**	111 (28.9)
Alcohol use, n (%)	24(9.7)	12 (8.8)	36 (9.4)	48 (12.5)
Hypertension, n (%)	127 (51.2)	81 (59.5)	208 (54.2)	200 (52.1)
Diabetes mellitus, n (%)	**15 (6.0)**	**9 (6.6)**	**24 (6.3)**	65 (16.9)
Hyperlipidemia, n (%)	**13 (5.2)**	**9 (6.6)**	**22 (5.7)**	8 (2.1)
Site of intracranial aneurysm, n (%)				
Internal carotid artery	113 (45.6)	174 (51.0)	287 (48.7)	–
Middle cerebral artery	45 (18.1)	69 (20.2)	114 (19.4)	–
Anterior cerebral artery	15 (6.0)	24 (7.0)	39 (6.6)	–
Posterior cerebral artery	2 (0.8)	13 (3.8)	15 (2.5)	–
Anterior communicating artery	41 (16.5)	20 (5.9)	61 (10.4)	–
Posterior communicating artery	18 (7.3)	16 (4.7)	34 (5.8)	–
Basilar/Vertebral artery	14 (5.6)	25 (7.3)	39 (5.1)	–

SD, standard deviation; Bold font indicates p < 0.05 compared with control group −, not available.

### Hardy-Weinberg Equilibrium and Linkage Disequilibrium Analysis

**Figure 1:**
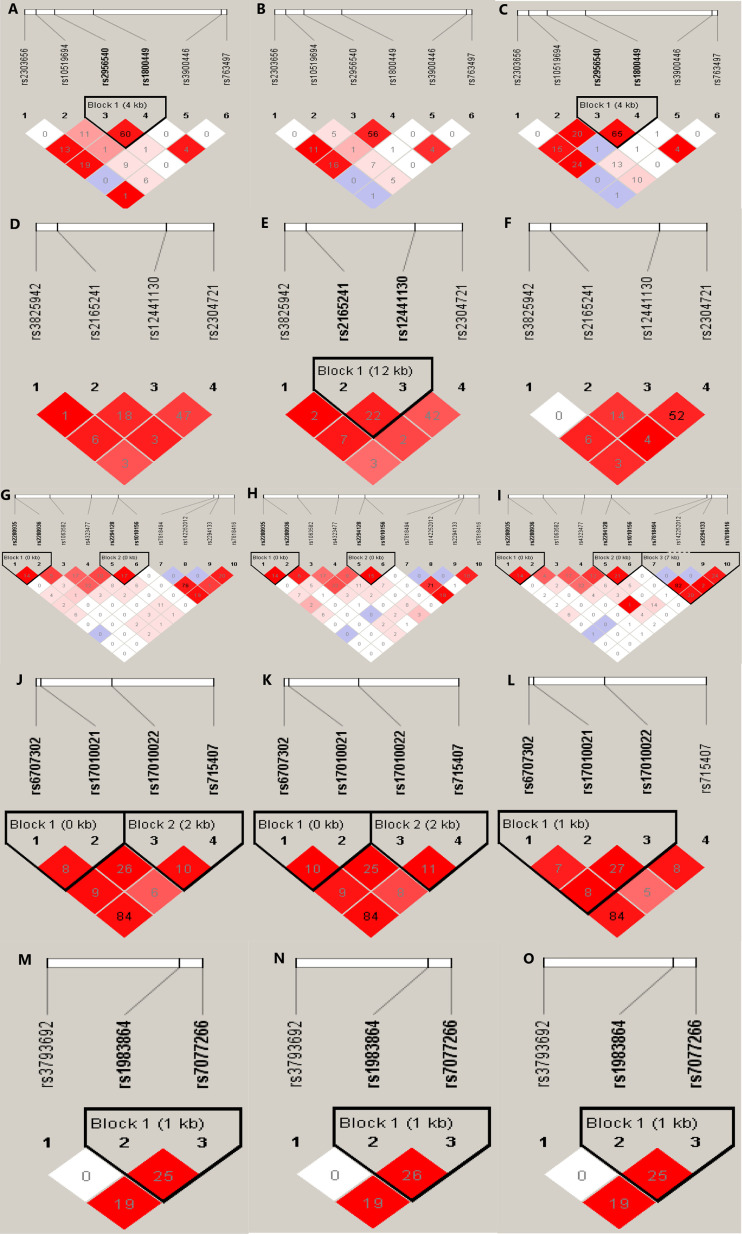
Graphical representation of the SNP locations and LD structure of each isoform of *LOX* family genes. The SNP distribution and haplotype block structure across *LOX* family genes are shown. The measures of LD (r^2^) among all possible pairs of SNPs are shown graphically according to the shade of color, where white represents very low r^2^ and scarlet represents very high r^2^. The numbers in squares are r^2^ values (r^2^ × 100). The graphics **(A–C)** represent the SNP locations and LD structures of *LOX* gene in total, IA and control group, respectively. Accordingly, graphics **(D–F)** were for *LOXL1*, graphics **(G–I)** were for *LOXL2*, graphics **(J–L)** were for *LOXL3* and graphic **(M–O)** were for *LOXL4*, respectively.

The detection rate of all SNP genotypes was 100%. Except for the allele frequency and genotype distribution of *LOXL2* rs2280935, all SNPs in the controls were distributed in Hardy-Weinberg equilibrium (*P* > 0.05; **Table 3**). **Figure 1** shows the linkage disequilibrium **(**LD) block of the targeted SNPs of the *LOX* family genes in all participants.

**Table 3:** Multivariate logistic regression analysis of association of polymorphisms in *LOX* family genes and risk of IA in Chinese population.

Gene	SNP	Genotype^a^			Dominant model		Recessive model		Additive model	
		Case (n)	Control (n)	P_HWE_^b^	OR (95%CI)	*P* value	OR (95%CI)	*P* value	OR (95%CI)	*P* value
*LOX*	rs1800449(C>T)	237/125/22	247/124/13	0.867	1.15(0.80-1.65)	0.450	1.52(0.65-3.56)	0.336	1.16(0.86-1.58)	0.328
	rs2956540(G>C)	195/147/42	210/146/28	0.931	1.28(0.90-1.83)	0.164	1.34(0.71-2.51)	0.365	1.23(0.93-1.61)	0.142
	rs10519694(C>T)	313/50/21	336/44/4	0.194	1.54(0.94-2.51)	0.084	**3.88(1.12-13.47)**	**0.033**	**1.56(1.05-2.34)**	**0.030**
	rs2303656(G>T)	348/36/0	342/41/1	0.981	0.72(0.40-1.30)	0.274	–	–	0.71(0.39-1.28)	0.250
	rs763497(A>G)	273/97/14	270/100/14	0.472	1.06(0.72-1.57)	0.753	0.95(0.39-2.34)	0.913	1.04(0.75-1.43)	0.824
	rs3900446(A>G)	305/72/7	313/66/5	0.778	1.01(0.65-1.58)	0.954	2.37(0.63-8.90)	0.200	1.09(0.74-1.61)	0.673
*LOXL1*	rs2165241(C>T)	303/76/5	313/66/5	0.780	1.06(0.68-1.65)	0.786	1.09(0.26-4.50)	0.909	1.06(0.71-1.57)	0.784
	rs3825942(G>A)	284/90/10	304/78/2	0.450	1.17(0.77-1.79)	0.458	1.85(0.30-11.28)	0.504	1.18(0.80-1.75)	0.398
	rs2304721(C>A)	223/133/28	208/147/29	0.910	1.11(0.77-1.58)	0.571	0.92(0.47-1.77)	0.792	1.05(0.80-1.38)	0.739
	rs12441130(T>C)	180/154/50	149/178/57	0.950	0.79(0.56-1.13)	0.202	0.94(0.57-1.54)	0.818	0.91(0.71-1.16)	0.907
*LOXL2*	rs2294128(C>T)	298/80/6	279/95/10	0.856	0.77(0.51-1.16)	0.209	0.62(0.17-2.26)	0.472	0.78(0.54-1.13)	0.186
	rs7818494(A>G)	241/121/22	236/129/19	0.969	0.97(0.68-1.40)	0.887	0.83(0.38-1.80)	0.632	0.96(0.71-1.28)	0.765
	rs4323477(A>G)	97/197/90	87/188/109	0.942	1.08(0.71-1.62)	0.731	0.67(0.45-1.01)	0.056	0.88(0.69-1.13)	0.327
	rs7818416(G>A)	119/190/75	122/178/84	0.458	0.92(0.63-1.33)	0.644	0.84(0.54-1.30)	0.428	0.91(0.72-1.16)	0.458
	rs1063582(G>T)	236/126/22	226/134/24	0.790	1.02(0.71-1.46)	0.922	1.08(0.51-2.29)	0.840	1.02(0.77-1.37)	0.875
	rs2280936(C>G)	241/125/18	245/124/15	0.990	0.98(0.68-1.41)	0.920	1.41(0.61-3.22)	0.420	1.03(0.76-1.39)	0.837
	rs2294133(C>T)	236/116/32	215/147/22	0.892	0.82(0.57-1.17)	0.276	1.39(0.69-2.78)	0.352	0.93(0.70-1.24)	0.627
	rs2280935(A>C)	155/177/52	128/212/44	0.007	0.77(0.53-1.10)	0.144	1.56(0.92-2.66)	0.102	0.96(0.74-1.26)	0.787
	rs1010156(T>C)	103/197/84	114/198/72	0.693	1.18(0.80-1.75)	0.401	1.07(0.69-1.66)	0.777	1.10(0.85-1.42)	0.473
	rs142252012(G>A)	373/11/0	372/12/0	0.953	1.05(0.36-3.05)	0.927	–	–	1.05(0.36-3.05)	0.927
*LOXL3*	rs715407(T>G)	252/121/11	278/98/8	0.983	1.29(0.89-1.89)	0.183	0.95(0.29-3.06)	0.931	1.22(0.87-1.72)	0.246
	rs6707302(C>T)	265/109/10	284/93/7	0.982	1.20(0.81-1.77)	0.360	0.92(0.26-3.25)	0.894	1.15(0.81-1.63)	0.431
	rs17010021(T>A)	166/176/42	167/178/39	0.702	1.04(0.73-1.48)	0.831	0.87(0.49-1.56)	0.642	0.99(0.76-1.30)	0.958
	rs17010022(C>G)	174/167/43	161/175/48	0.999	0.99(0.69-1.42)	0.982	0.83(0.48-1.45)	0.516	0.96(0.74-1.24)	0.742
*LOXL4*	rs3793692(G>A)	86/206/92	91/198/95	0.828	0.84(0.57-1.26)	0.410	0.97(0.65-1.47)	0.894	0.93(0.72-1.19)	0.555
	rs1983864(G>T)	126/192/66	124/183/77	0.818	1.05(0.72-1.52)	0.817	0.73(0.46-1.15)	0.172	0.93(0.72-1.19)	0.549
	rs7077266(G>T)	271/104/9	273/97/14	0.358	1.02(0.70-1.50)	0.911	0.81(0.31-2.16)	0.682	0.99(0.72-1.37)	0.964

SNP, single nucleotide polymorphism; OR, odds ratio; CI, confidence interval; −, not available.

^a^Genotype presented as wild type/heterozygous/homozygous; ^b^Hardy-Weinberg equilibrium test; Bold font indicates p < 0.05.

### Associations Between *LOX* Family Genes Polymorphisms and IA Susceptibility

**Table 3** lists the genotypes of 27 SNP of *LOX* family genes and their associations with IA. The results of univariate logistic regression analyses associated *LOX* rs10519694, *LOXL1* rs3825942, and *LOXL3* rs715407 polymorphisms with risk of total IA (**Table S1**). However, only the association of *LOX* rs10519694 and IA remained significant after adjusting for age, smoking status, diabetes mellitus, and hyperlipidemia (recessive and additive models: OR, 3.88; 95%CI, 1.12–13.47; *p* = 0.033; and OR, 1.56; 95%CI, 1.05–2.34; *p* = 0.030, respectively; **Table 3**). The calculated statistical power of the association of *LOX* rs10519694 and risk of IA under recessive and additive model was 100% and 41%, respectively. The association was verified when used the 208 Chinese Han populations in the 1000 Genome Project as additional controls under additive model (OR, 1.89; 95%CI, 1.24–2.89; *p* = 0.003) (**Table S2**).

### Stratification Analysis

Considering that single IA and multiple IA might be formed *via* different mechanisms, we stratified the patients according to the number of IA to assess associations between variants of *LOX* family genes and risk of single or multiple IA. Only the *LOX* gene polymorphism was associated with the risk of single IA after adjusting for age, smoking status, diabetes mellitus, and hyperlipidemia. The SNP of *LOX* rs10519694 was associated with an increased risk of single IA (recessive and additive models: OR, 3.95; 95%CI, 1.04–15.11, P = 0.044; SP, 100% and OR, 1.64; 95%CI, 1.04–2.56, P = 0.032, SP, 42%, respectively, **Table 4**). The association was verified when used the 208 additional population control under additive model (OR, 1.97; 95%CI, 1.26–3.09, P = 0.003) (**Table S3**). Another SNP of *LOX* rs2303656 was found to be associated with a decreased risk of single IA (dominant and additive models: OR, 0.44; 95%CI, 0.19–0.96; P = 0.041 and OR, 0.44, 95%CI, 0.20–0.96; P = 0.04, respectively, **Table 4**). However, the statistical power was relatively low (60%) and the association was not verified in the additional population control. Another gene polymorphism *LOXL2* rs1010156 SNP was found to be positively associated with risk of multiple IA in the dominant model after adjusting for potential confounders (OR, 1.92; 95% CI, 1.02–3.62, P = 0.044; **Table 5**) and the SP was 90% although the association was not replicated in the additional control (**Table S4**).

**Table 4:** Multivariate logistic regression analysis of associations of polymorphisms in *LOX* family genes and risk of single IA in a Chinese population.

GENE	SNP	Genotype^a^		Dominant model		Recessive model		Additive model	
		Case (n)	Control (n)	OR (95%CI)	*P* value	OR (95%CI)	*P* value	OR (95%CI)	*P* value
*LOX*	rs1800449(C>T)	163/72/13	247/124/13	0.99(0.65-1.51)	0.972	1.31(0.48-3.58)	0.594	1.03(0.72-1.47)	0.876
	rs2956540(G>C)	137/87/24	210/146/28	1.11(0.74-1.67)	0.615	1.21(0.58-2.51)	0.617	1.10(0.80-1.51)	0.546
	rs10519694(C>T)	201/32/15	336/44/4	1.64(0.95-2.83)	0.078	**3.95(1.04-15.11)**	**0.044**	**1.64(1.04-2.56)**	**0.032**
	rs2303656(G>T)	233/15/0	342/41/1	**0.44(0.19-0.96)**	**0.041**	–	–	**0.44(0.20-0.96)**	**0.040**
	rs763497(A>G)	177/64/7	270/100/14	0.79(0.27-2.35)	0.672	1.01(0.65-1.58)	0.954	0.98(0.68-1.42)	0.919
	rs3900446(A>G)	203/40/5	313/66/5	0.86(0.51-1.44)	0.560	3.64(0.92-14.34)	0.065	1.01(0.64-1.59)	0.969
*LOXL1*	rs2165241(C>T)	201/43/4	313/66/5	0.98(0.59-1.65)	0.952	1.30(0.28-6.16)	0.739	1.01(0.64-1.59)	0.966
	rs3825942(G>A)	186/56/6	304/78/2	1.07(0.66-1.73)	0.792	2.29(0.32-16.38)	0.410	1.11(0.71-1.73)	0.656
	rs2304721(C>A)	141/84/23	208/147/29	1.18(0.79-1.78)	0.420	1.18(0.58-2.41)	0.652	1.14(0.83-1.55)	0.420
	rs12441130(T>C)	116/97/35	156/171/57	0.85(0.56-1.27)	0.424	1.19(0.69-2.06)	0.525	0.97(0.73-1.28)	0.820
*LOXL2*	rs2294128(C>T)	198/54/4	279/95/10	0.70(0.44-1.14)	0.152	0.43(0.08-2.21)	0.309	0.71(0.46-1.09)	0.117
	rs7818494(A>G)	158/74/16	236/129/19	0.91(0.60-1.38)	0.664	0.99(0.42-2.33)	0.976	0.94(0.67-1.32)	0.718
	rs4323477(A>G)	60/129/59	89/186/109	1.13(0.70-1.83)	0.616	0.72(0.45-1.14)	0.159	0.92(0.69-1.22)	0.559
	rs7818416(G>A)	92/110/46	125/175/84	0.89(0.58-1.36)	0.586	0.72(0.43-1.21)	0.216	0.86(0.65-1.14)	0.297
	rs1063582(G>T)	154/85/9	226/134/24	1.10(0.73-1.66)	0.647	0.84(0.34-2.09)	0.714	1.01(0.74-1.46)	0.816
	rs2280936(C>G)	153/85/10	245/124/15	1.03(0.68-1.56)	0.880	1.11(0.39-3.13)	0.848	1.04(0.73-1.48)	0.846
	rs2294133(C>T)	148/74/26	215/147/22	0.88(0.58-1.32)	0.530	1.88(0.89-3.96)	0.096	1.03(0.75-1.42)	0.839
	rs2280935(A>C)	101/111/36	140/200/44	0.88(0.58-1.33)	0.531	1.58(0.86-2.91)	0.141	1.04(0.76-1.42)	0.795
	rs1010156(T>C)	75/118/55	114/198/72	0.94(0.60-1.46)	0.780	0.95(0.57-1.60)	0.855	0.96(0.72-1.28)	0.772
	rs142252012(G>A)	241/7/0	372/12/0	1.20(0.34-4.27)	0.780	–	–	1.20(0.34-4.27)	0.780
*LOXL3*	rs715407(T>G)	161/79/8	278/98/8	1.22(0.79-1.90)	0.366	1.18(0.32-4.36)	0.800	1.19(0.81-1.75)	0.381
	rs6707302(C>T)	168/73/7	284/93/7	1.18(0.75-1.85)	0.475	1.12(0.26-4.69)	0.880	1.15(0.77-1.72)	0.494
	rs17010021(T>A)	112/111/25	167/178/39	0.87(0.58-1.30)	0.493	0.78(0.39-1.54)	0.468	0.87(0.64-1.19)	0.392
	rs17010022(C>G)	110/112/26	161/175/48	0.11(0.74-1.68)	0.609	0.85(0.45-1.61)	0.614	1.02(0.75-1.38)	0.892
*LOXL4*	rs3793692(G>A)	53/135/60	94/195/95	0.98(0.61-1.57)	0.930	0.99(0.62-1.59)	0.962	0.99(0.84-1.32)	0.933
	rs1983864(G>T)	73/127/48	129/178/77	1.06(0.69-1.63)	0.791	0.77(0.45-1.31)	0.334	0.95(0.71-1.26)	0.717
	rs7077266(G>T)	170/73/5	273/97/14	1.02(.66-1.59)	0.917	0.73(0.22-2.45)	0.615	0.99(0.68-1.44)	0.938

SNP, single nucleotide polymorphism; OR, odds ratio; CI, confidence interval; −, not available.

^a^Genotype presented as wild type/heterozygous/homozygous; Bold font indicates p < 0.05.

**Table 5:** Univariate logistic regression analysis of association of polymorphisms in *LOX* family genes and risk of multiple IAs in a Chinese population.

GENE	SNP	Genotype^a^		Dominant model		Recessive model		Additive model	
		Case (n)	Control (n)	OR (95%CI)	*P* value	OR (95%CI)	*P* value	OR (95%CI)	*P* value
*LOX*	rs1800449(C>T)	74/53/9	247/124/13	1.41(0.84-2.38)	0.198	1.77(0.58-5.38)	0.316	1.37(0.89-2.11)	0.150
	rs2956540(G>C)	58/60/18	210/206/46	1.61(0.95-2.70)	0.075	1.59(0.67-3.78)	0.294	1.44(0.97-2.13)	0.070
	rs10519694(C>T)	112/18/6	336/44/4	1.48(0.71-3.09)	0.297	4.28(0.76-24.08)	0.099	1.52(0.82-2.80)	0.184
	rs2303656(G>T)	115/21/0	342/41/1	**-**	**-**	1.22(0.56-2.65)	0.614	1.19(0.56-2.55)	0.647
	rs763497(A>G)	96/33/7	270/100/14	1.23(0.3-4.17)	0.741	1.13(0.65-1.98)	0.669	1.12(0.71-1.76)	0.638
	rs3900446(A>G)	102/32/2	313/66/5	1.39(0.75-2.59)	0.299	0.65(0.04-9.88)	0.755	1.30(0.73-2.31)	0.374
*LOXL1*	rs2165241(C>T)	102/33/1	313/66/5	1.12(0.60-2.10)	0.722	0.66(0.07-6.3)	0.711	1.06(0.61-1.86)	0.834
	rs3825942(G>A)	98/34/4	304/78/2	1.36(0.74-2.50)	0.315	1.11(0.11-10.41)	0.931	1.31(0.75-2.29)	0.349
	rs2304721(C>A)	82/49/5	208/147/29	1.10(0.65-1.85)	0.730	0.52(0.14-1.88)	0.315	0.97(0.64-1.49)	0.904
	rs12441130(T>C)	64/57/15	156/171/57	0.84(0.50-1.42)	0.512	0.62(0.28-1.39)	0.250	0.82(0.56-1.19)	0.297
*LOXL2*	rs2294128(C>T)	100/34/2	279/95/10	0.97(0.54-1.76)	0.922	1.10(0.20-5.96)	0.909	0.99(0.59-1.66)	0.959
	rs7818494(A>G)	83/47/6	236/129/19	1.08(0.64-1.92)	0.786	0.66(1.19-2.33)	0.519	0.99(0.64-1.54)	0.984
	rs4323477(A>G)	39/66/31	89/186/109	0.92(0.51-1.67)	0.793	0.92(0.51-1.67)	0.793	0.56(0.30-1.06)	0.075
	rs7818416(G>A)	51/56/29	125/175/84	1.01(0.58-1.74)	0.977	1.13(0.61-2.10)	0.691	1.04(0.74-1.48)	0.809
	rs1063582(G>T)	82/41/13	226/134/24	0.90(0.52-1.53)	0.684	1.80(0.65-5.04)	0.260	1.02(0.66-1.58)	0.928
	rs2280936(C>G)	88/40/8	245/124/15	0.85(0.49-1.45)	0.542	2.04(0.72-5.83)	0.182	1.00(0.65-1.55)	1.000
	rs2294133(C>T)	88/42/6	215/147/22	0.73(0.43-1.24)	0.248	0.62(1.18-2.14)	0.447	0.75(0.48-1.18)	0.215
	rs2280935(A>C)	63/57/16	140/200/44	0.62(0.37-1.05)	0.077	1.47(0.68-3.18)	0.324	0.84(0.56-1.26)	0.398
	rs1010156(T>C)	28/29/79	114/198/72	**1.92(1.02-3.62)**	**0.044**	1.31(0.69-2.49)	0.403	1.42(0.97-2.10)	0.073
	rs142252012(G>A)	132/4	372/12/0	0.94(0.21-4.12)	0.932	–	–	0.94(0.21-4.12)	0.932
*LOXL3*	rs715407(T>G)	91/42/3	278/98/8	1.46(0.84-2.55)	0.177	0.64(0.09-4.42)	0.649	1.32(0.80-2.16)	0.277
	rs6707302(C>T)	97/36/3	284/93/7	1.29(0.73-2.27)	0.376	0.68(0.09-4.92)	0.702	1.20(0.72-1.99)	0.489
	rs17010021(T>A)	55/64/17	167/178/39	1.35(0.79-2.30)	0.269	1.12(0.49-2.53)	0.794	1.21(0.82-1.79)	0.339
	rs17010022(C>G)	64/55/17	161/175/48	0.74(0.44-1.24)	0.252	0.85(0.38-1.93)	0.705	0.82(0.55-1.21)	0.306
*LOXL4*	rs3793692(G>A)	36/68/32	94/195/95	0.76(0.44-1.34)	0.347	0.99(0.54-1.85)	0.990	0.90(0.62-1.29)	0.550
	rs1983864(G>T)	54/64/18	129/178/77	0.93(0.54-1.59)	0.777	0.71(0.35-1.43)	0.331	0.87(0.61-1.26)	0.471
	rs7077266(G>T)	101/31/4	273/97/14	0.88(0.49-1.55)	0.647	1.10(0.30-3.98)	0.887	0.92(0.57-1.48)	0.742

SNP, single nucleotide polymorphism; OR, odds ratio; CI, confidence interval; −, not available.

^a^Genotype presented as wild type/heterozygous/homozygous; Bold font indicates p < 0.05.

## Discussion

We aimed to determine associations between polymorphisms of *LOX* family genes and risk of IA in a Chinese population. We found that the *LOX* rs10519694 and *LOXL2* rs1010156 polymorphism was associated with the risk of single and multiple IA respectively. As far as we can ascertain, this is the first systematic exploration of associations between *LOX* family genes polymorphisms and the risk of IA in a Chinese population, and the findings offer a new perspective for the prevention and treatment of IA.

The *LOX* family genes comprise five members which encode LOX and LOX–like isoenzymes (LOXL1, LOXL2, LOXL3, and LOXL4), which are copper-dependent oxidases with a conserved C-terminal region that corresponds to the catalytic domain and a different N-termini (29). Therefore, each isoform has similar amine oxidase activities in which the ϵ-amino group of peptidyl lysine is oxidized to generate peptidyl aldehyde (20, 30, 31). Collagen and elastin are the specific physiological substrates of LOX family oxidases. Cross-linked collagen and elastic fibers are essential for ECM stability and provide most of the tensile strength and structural integrity of connective tissue (32). The abnormal expression of LOX enzymes is related to changes in ECM composition characteristic of atherosclerosis and aortic aneurysms (21).

The *LOX* located at 5q23.1, which was the first identified subtype and represent of the *LOX* family genes.LOX catalyzes the translation of peptidyl lysine and oxidizes it to peptidyl aldehyde and α–aminohexyl–δ semialdehyde. The collagen and elastin crosslinking initiated by this chemical reaction assures deposition of these fiber proteins in the ECM (33). Changes in the ECM disrupt the vascular wall, as evidenced in atherosclerosis, a cardiovascular disease that features vigorous destruction of the ECM (34, 35). Some morphological changes caused by atherosclerosis can result in the deposition of fibrous tissues that ultimately develop into IA (13, 36). Thus, we hypothesis that the disrupted LOX expression might be associated with IA that is characterized by ECM destruction. The present study found that *LOX* rs10519694 was associated with the increased risk of single IA in recessive and additive models and the association was verified in the additional population control. Furthermore, similar to the findings of a case-control study by Hong et al., who associated the A allele of another polymorphism of *LOX* (rs2303656) with decreased risk of IA (*P* = 8.2 × 10^-4^) in Korean population, we also identified that this variant was associated with the decreased risk of single IA in Chinese, even a definite conclusion couldn’t be drawn considering the fact of that the statistical power was relatively low and the association was not replicated in the additional control. Hong et al. also found that the C allele of *LOX* rs3900446 and the G allele of rs763497 with increased risk of IA (OR, 20.15; *P* = 4.8 × 10^-5^ and OR, 2.26; *P* = 4.8 × 10^-5^) in Korean population, respectively (25). However, the polymorphisms of *LOX* gene is not associated with IA in Japanese and Dutch (23), central European (22) and South Indian (24) populations. Lan Ma et al. found that another SNP of *LOX* (rs1800449) was associated with the susceptibility to coronary artery diseases in Chinese population (37) and a recent mouse model also suggested that the missense mutation (*p*.G473A) caused by rs1800449 could lead to loss tumor suppressor function of the LOX propeptide and thus accelerate carcinogen-induced tumor formation (38). However, this SNP was not found to be associated with IA in our study. We speculate that these results are associated with the population genetic heterogeneity or limited statistical power of previous studies. These lines of evidence illustrated that the polymorphisms of *LOX* may perform roles in IA susceptibility in particular populations. The detail functions of the identified variants were not clear, considering the fact of that rs10519694 and rs2303656 are both intronic variants, as a tagSNP, the associations between them and IA might exist different possibilities: the polymorphism has a causal role or the polymorphism has no causal role but is associated with a nearby causal variant. The detailed mechanism of *LOX* polymorphisms on IA should be further explored.

*LOXL1–4* are located at 15q24.1, 8p21.3, 2p13.1, and 10q24.2, respectively. Similar with LOX, LOXL1 also contains an N-terminal propeptide, while the N-terminal of LOXL2-4 consists of four scavenger receptor cysteine-rich (SRCR) domains (29). The biological effects of LOXL1 are similar to those of LOX, and are significant not only for elastogenesis but also for supporting the deposition of elastin (39). For example, a large-scale GWAS study found that *LOXL1* variants can increase the deposition of elastin and fibrillin-1 that stabilizes the ECM, thereby protecting against exfoliation syndrome (40). Since LOXL2 affects the pathophysiological processes of cancer, such as cell adhesion and invasion (30) and promotes endothelial tube formation by cross–linking collagen IV in the vascular system, its expression may be related to tumor angiogenesis and progression (21). The glycosylation and proteolytic processing of extracellular LOXL2 are essential for cross–linking basement membrane type IV collagen in the ECM (41, 42). A recent crystal structure study found that a point mutation of LOXL2 (N455Q) could affect the activity of the catalytic domain by eliminating the glycosylation in the fourth SRCR domains of LOXL2, and another mutation (R257G) could prevent LOXL2 from proteolytic cleavage by serine protease, resulting in LOXL2 couldn’t remove the first two SRCR domains and subsequently can’t bind to type IV collagen, finally disturb the structural and functional stability of ECM (29, 41). LOXL3 is expressed abundantly in the retina and central nervous system; abnormal LOXL3 expression is associated with cleft palate and spinal deformities (43). These anomalies may be associated with the inability of LOXL3 to effectively crosslink collagen (30). The *LOXL4* is the latest discovery in the LOX family and limited information is available regarding this isoform. However, LOXL4 plays specific roles in the proliferation and metastasis of cells in certain malignancies, such as gastric and liver cancer (44, 45). Considering the role of LOXL4 in tumorigenesis, it may serve as a potential independent prognostic marker and therapeutic target for these cancers. Further detailed investigations are necessary to fully understand the biological functions of *LOX* family gene members.

We found that one SNP of *LOXL2* rs1010156 associated with the risk of multiple IA after adjusting for potential confounders (OR, 1.92; 95% CI, 1.02–3.62, P = 0.044). Even the result was not verified in the additional control, we thought that the previous analysis was more reasonable due to that we just performed univariate logistic regression analysis in the additional controls and potential confounders were not adjusted due to these informations was not available. Our findings were similar to the results obtained by Akagawa et al. (18) who systematically screened *LOXL* family genes and reported that the *LOXL2* polymorphism was associated with susceptibility to FIA. However, a comparison of SIA and FIA between patients and controls did not reveal any associations. In addition, using WES, Wu et al. found that a rare variant of *LOXL2* c.133C>T (rs142252012) can increase susceptibility to FIA (26), however, we could not verify the association between this variant and susceptibility to IA in Chinese patients with SIA. We found an association between *LOXL2* and multiple IA but not single IA, which may be attributed to the involvement of different pathophysiological pathways in the formation of multiple and single IA. Unlike single IA, multiple IA were not distributed randomly in the Circle of Willis, but were rather arranged in clusters near the index aneurysm, which may predict the formation of mirror aneurysms. Such aneurysmal clusters may be affected by genetic and hemodynamic variables (46). Multiple IA that develop more frequently in patients with familial IA can be larger and rupture at a higher frequency in younger individuals (14), which further suggests a genetic predisposition. This study did not find an association between *LOXL1*, *LOXL3*, and *LOXL4* polymorphisms and susceptibility to IA, which was consistent with the results of Akagawa et al., who found no associations between *LOXL1*, *LOXL3*, and *LOXL4* gene polymorphisms and IA susceptibility in a Japanese population (18). Considering the limited power of the statistics, larger sample size studies are needed in the future.

This study has several limitations. First, the controls comprised individuals who had not been diagnosed with IA or other cerebrovascular diseases. As some participants were assessed by imaging modalities, classification might have been biased. However, this influence was probably small considering that few people had IA in China. Second, we explored associations between polymorphisms in LOX family genes and risk of single and multiple IAs. Due to the sample size was relatively small and the statistical power was limited, the results may be not stable, further studies with larger sample size are needed to verify our findings. Third, considering the fact that the identified associated variants were tagSNP of target genes, we didn’t know whether these variants were the real causal ones or just the surrogate of nearby causal variants, the potential functions and the detail pathological mechanisms of polymorphisms of *LOX* family genes on IA are needed to further explored. Furthermore, this study has been performed in a central south Chinese population and the results may can’t directly extrapolate to other ethnicities considering the fact that there may be population genetic heterogeneity. Our results need to be validated in further studies. Nevertheless, this study is the first to reveal associations between polymorphisms of the *LOX* family genes and susceptibility to IA in the Chinese population and offers a new perspective for the early diagnosis of IA.

## Conclusions

Our findings showed that *LOX* and *LOXL2* polymorphisms were associated with risk of single IA and multiple IA in a Chinese population, suggesting potential roles of these genes in IA. Further investigations are imperative to elucidate the effects of these genes on IA.

## Data Availability Statement

The original contributions presented in the study are included in the article/**Supplementary Material**. Further inquiries can be directed to the corresponding author.

## Ethics Statement

The studies involving human participants were reviewed and approved by The Ethics Committee at Central South University (Permit No: CTXY–150002–1). The patients/participants provided their written informed consent to participate in this study.

## Author Contributions

Conceptualization: CL, CH, and JY. Data curation: CL, BL, and JL. Formal analysis: CL and BL. Funding acquisition: JY. Investigation: CL, CH, BL, JL, LH, RD, XL, JZ, LX, SL, YL, DY, and WJ. Methodology: CH, CL, and JY. Project administration: CL, BL, and JY. Resources: CH, JL, WJ, and JY. Supervision: CL, CH, and JY. Writing—original draft preparation: CL. Writing—review and editing: JY. All authors contributed to the article and approved the submitted version.

## Funding

This work was supported by the National Nature Science Foundation, China [grant numbers: 81502881], and the Graduate Student Innovative Scientific Research Project of Central South University, China [grant numbers:1053320192459].

## Conflict of Interest

The authors declare that the research was conducted in the absence of any commercial or financial relationships that could be construed as a potential conflict of interest.

## Publisher’s Note

All claims expressed in this article are solely those of the authors and do not necessarily represent those of their affiliated organizations, or those of the publisher, the editors and the reviewers. Any product that may be evaluated in this article, or claim that may be made by its manufacturer, is not guaranteed or endorsed by the publisher.
