# Supplementary material for: Polymorphisms in Lysyl Oxidase Family Genes Are Associated With Intracranial Aneurysm Susceptibility in a Chinese Population
Source: Front Endocrinol (Lausanne). 2021 Jul 28;12:642698. doi: 10.3389/fendo.2021.642698 (PMC8355735; doi:10.3389/fendo.2021.642698)
Supplement: Supplementary file 1 [file DataSheet_1.docx]

| **Supplementary Table 1. Univariate logistic regression analysis of associations of polymorphisms in *LOX* family genes and risk of IA in a Chinese population** | | | | | | | | | | | | | |
| --- | --- | --- | --- | --- | --- | --- | --- | --- | --- | --- | --- | --- | --- |
| GENE | SNP | Genotype^a^ | | *P*_HWE_^b^ |  | Dominant model | |  | Recessive model | |  | Additive model | |
|  |  | Case (n) | Control (n) |  |  | OR（95%CI） | *P* value |  | OR（95%CI） | *P* value |  | OR（95%CI） | *P* value |
| *LOX* | rs1800449(C>T) | 237/125/22 | 247/124/13 | 0.867 |  | 1.12(0.83-1.50) | 0.455 |  | 1.73(0.86-3.50) | 0.124 |  | 1.16(0.91-1.48) | 0.236 |
|  | rs2956540(G>C) | 195/147/42 | 210/146/28 | 0.931 |  | 1.17(0.88-1.55) | 0.278 |  | 1.56(0.95-2.58) | 0.081 |  | 1.19(0.96-1.48) | 0.110 |
|  | rs10519694(C>T) | 313/50/21 | 336/44/4 | 0.194 |  | **1.59(1.07-2.36)** | **0.023** |  | **5.50(1.87-16.17)** | **0.002** |  | **1.64(1.19-2.27)** | **0.002** |
|  | rs2303656(G>T) | 348/36/0 | 342/41/1 | 0.981 |  | 0.84(0.53-1.35) | 0.474 |  | - | - |  | 0.83(0.52-1.31) | 0.413 |
|  | rs763497(A>G) | 273/97/14 | 270/100/14 | 0.472 |  | 0.96(0.71-1.31) | 0.812 |  | 1.00(0.47-2.13) | 1.000 |  | 0.97(0.75-1.26) | 0.842 |
|  | rs3900446(A>G) | 305/72/7 | 313/66/5 | 0.778 |  | 1.14(0.80-1.63) | 0.467 |  | 1.41(0.44-4.47) | 0.562 |  | 1.14(0.83-1.57) | 0.417 |
|  |  |  |  |  |  |  |  |  |  |  |  |  |  |
| *LOXL1* | rs2165241(C>T) | 303/76/5 | 313/66/5 | 0.780 |  | 1.18(0.83-1.68) | 0.365 |  | 1.00(0.29-3.48) | 1.000 |  | 1.15(0.83-1.58) | 0.411 |
|  | rs3825942(G>A) | 284/90/10 | 304/78/2 | 0.450 |  | 1.34(0.96-1.87) | 0.089 |  | **5.11(1.11-23.46)** | **0.036** |  | **1.40(1.03-1.90)** | **0.032** |
|  | rs2304721(C>A) | 223/133/28 | 208/147/29 | 0.910 |  | 0.85(0.64-1.14) | 0.276 |  | 0.96(0.56-1.65) | 0.891 |  | 0.90(0.72-1.13) | 0.361 |
|  | rs12441130(T>C) | 180/154/50 | 156/171/57 | 0.950 |  | 0.78(0.58-1.03) | 0.081 |  | 0.86(0.57-1.29) | 0.466 |  | 0.85(0.69-1.04) | 0.110 |
|  |  |  |  |  |  |  |  |  |  |  |  |  |  |
| *LOXL2* | rs2294128(C>T) | 298/80/6 | 279/95/10 | 0.856 |  | 0.77(0.55-1.07) | 0.113 |  | 0.59(0.21-1.65) | 0.317 |  | 0.78(0.58-1.04) | 0.090 |
|  | rs7818494(A>G) | 241/121/22 | 236/129/19 | 0.969 |  | 0.95(0.71-1.27) | 0.710 |  | 1.17(0.62-2.19) | 0.630 |  | 0.99(0.78-1.25) | 0.903 |
|  | rs4323477(A>G) | 97/197/90 | 87/188/109 | 0.942 |  | 0.87(0.62-1.21) | 0.402 |  | 0.77(0.56-1.07) | 1.118 |  | 0.86(0.70-1.05) | 0.141 |
|  | rs7818416(G>A) | 119/190/75 | 122/178/84 | 0.458 |  | 0.81(0.60-1.10) | 0.173 |  | 0.87(0.61-1.23) | 0.423 |  | 0.88(0.72-1.06) | 0.184 |
|  | rs1063582(G>T) | 236/126/22 | 226/134/24 | 0.790 |  | 0.90(0.67-1.20) | 0.461 |  | 0.91(0.50-1.66) | 0.761 |  | 0.92(0.73-1.16) | 0.476 |
|  | rs2280936(C>G) | 241/125/18 | 245/124/15 | 0.990 |  | 1.05(0.78-1.40) | 0.765 |  | 1.21(0.60-2.44) | 0.594 |  | 1.06(0.83-1.35) | 0.659 |
|  | rs2294133(C>T) | 236/116/32 | 215/147/22 | 0.892 |  | 0.80(0.60-1.06) | 0.124 |  | 1.50(0.85-2.63) | 0.160 |  | 0.93(0.74-1.17) | 0.525 |
|  | rs2280935(A>C) | 155/177/52 | 128/212/44 | 0.007 |  | 0.77(0.58-1.03) | 0.077 |  | 1.21(0.79-1.86) | 0.383 |  | 0.91(0.74-1.13) | 0.388 |
|  | rs1010156(T>C) | 103/197/84 | 114/198/72 | 0.693 |  | 1.15(0.84-1.58) | 0.378 |  | 1.21(0.85-1.73) | 0.282 |  | 1.13(0.92-1.39) | 0.231 |
|  | rs142252012(G>A) | 373/11/0 | 372/12/0 | 0.953 |  | 0.91(0.40-2.10) | 0.832 |  | - | - |  | 0.91(0.40-2.10) | 0.832 |
|  |  |  |  |  |  |  |  |  |  |  |  |  |  |
| *LOXL3* | rs715407(T>G) | 252/121/11 | 278/98/8 | 0.983 |  | **1.37(1.01-1.87)** | **0.043** |  | 1.39(0.55-3.49) | 0.488 |  | **1.32(1.01-1.74)** | **0.045** |
|  | rs6707302(C>T) | 265/109/10 | 284/93/7 | 0.982 |  | 1.28(0.93-1.75) | 0.129 |  | 1.44(0.54-3.82) | 0.464 |  | 1.25(0.95-1.66) | 0.118 |
|  | rs17010021(T>A) | 166/176/42 | 167/178/39 | 0.702 |  | 1.01(0.76-1.35) | 0.942 |  | 1.09(0.69-1.72) | 0.725 |  | 1.02(0.82-1.26) | 0.869 |
|  | rs17010022(C>G) | 174/167/43 | 161/175/48 | 0.999 |  | 0.87(0.66-1.16) | 0.344 |  | 0.88(0.57-1.37) | 0.577 |  | 0.90(0.73-1.11) | 0.335 |
|  |  |  |  |  |  |  |  |  |  |  |  |  |  |
| *LOXL4* | rs3793692(G>A) | 86/206/92 | 91/198/95 | 0.828 |  | 1.07(0.77-1.50) | 0.672 |  | 0.96(0.69-1.33) | 0.801 |  | 1.01(0.82-1.24) | 0.916 |
|  | rs1983864(G>T) | 126/192/66 | 124/183/77 | 0.818 |  | 1.02(0.75-1.38) | 0.878 |  | 0.83(0.58-1.19) | 0.308 |  | 0.95(0.78-1.17) | 0.645 |
|  | rs7077266(G>T) | 271/104/9 | 273/97/14 | 0.358 |  | 1.03(0.75-1.40) | 0.874 |  | 0.63(0.27-1.48) | 0.294 |  | 0.97(0.74-1.27) | 0.837 |

SNP, single nucleotide polymorphism; OR, odds ratio; CI, confidence interval; HWE, Hardy-Weinberg equilibrium; −, not available. ^a^Genotype presented as wild type/heterozygous/homozygous，^b^HWE p value for the control group; Bold font indicates p<0.05.

| **Supplementary Table 2. Univariate logistic regression analysis of associations of polymorphisms in *LOX* family genes and risk of IA in the additional Chinese population control** | | | | | | | | | | | | |
| --- | --- | --- | --- | --- | --- | --- | --- | --- | --- | --- | --- | --- |
| GENE | SNP | Genotype^a^ | |  | Dominant model | |  | Recessive model | |  | Additive model | |
|  |  | Case (n) | Control (n)^b^ |  | OR (95%CI) | *P* value |  | OR (95%CI) | *P* value |  | OR (95%CI) | *P* value |
| *LOX* | rs1800449(C>T) | 237/125/22 | 132/64/12 |  | 1.08(0.76-1.53) | 0.676 |  | 0.99(0.48-2.05) | 0.984 |  | 1.05(0.79-1.39) | 0.742 |
|  | rs2956540(G>C) | 195/147/42 | - |  | - | - |  | - | - |  | - | - |
|  | rs10519694(C>T) | 313/50/21 | 184/24/0 |  | **1.74(1.06-2.86)** | **0.029** |  | - | - |  | **1.89(1.24-2.89)** | **0.003** |
|  | rs2303656(G>T) | 348/36/0 | 192/15/1 |  | 1.24(0.67-2.30) | 0.491 |  | - | - |  | 1.16(0.64-2.09) | 0.632 |
|  | rs763497(A>G) | 273/97/14 | 150/50/8 |  | 1.05(0.72-1.53) | 0.793 |  | 0.95(0.39-2.29) | 0.902 |  | 1.03(0.75-1.41) | 0.860 |
|  | rs3900446(A>G) | 305/72/7 | 179/28/1 |  | **1.60(1.01-2.54)** | **0.047** |  | 3.84(0.47-31.45) | 0.209 |  | **1.60(1.04-2.45)** | **0.033** |
|  |  |  |  |  |  |  |  |  |  |  |  |  |
| *LOXL1* | rs2165241(C>T) | 303/76/5 | 178/29/1 |  | **1.59(1.00-2.51)** | **0.048** |  | 2.73(0.32-23.53) | 0.361 |  | **1.57(1.02-2.41)** | **0.041** |
|  | rs3825942(G>A) | 284/90/10 | 157/46/5 |  | 1.08(0.73-1.60) | 0.685 |  | 1.09(0.37-3.22) | 0.882 |  | 1.07(0.76-1.50) | 0.691 |
|  | rs2304721(C>A) | 223/133/28 | 115/77/16 |  | 0.89(0.64-1.26) | 0.514 |  | 0.94(0.50-1.79) | 0.859 |  | 0.92(0.71-1.21) | 0.558 |
|  | rs12441130(T>C) | 180/154/50 | 93/85/30 |  | 0.92(0.65-1.29) | 0.614 |  | 0.89(0.55-1.45) | 0.634 |  | 0.93(0.73-1.18) | 0.554 |
|  |  |  |  |  |  |  |  |  |  |  |  |  |
| *LOXL2* | rs2294128(C>T) | 298/80/6 | 142/63/3 |  | **0.62(0.43-0.91)** | **0.013** |  | 1.09(0.27-4.38) | 0.909 |  | **0.68(0.48-0.95)** | **0.026** |
|  | rs7818494(A>G) | 241/121/22 | 127/76/5 |  | 0.93(0.66-1.32) | 0.683 |  | 2.47(0.92-6.62) | 0.073 |  | 1.05(0.78-1.41) | 0.745 |
|  | rs4323477(A>G) | 97/197/90 | 51/97/60 |  | 0.96(0.65-1.42) | 0.842 |  | 0.76(0.52-1.11) | 0.149 |  | 0.89(0.70-1.12) | 0.314 |
|  | rs7818416(G>A) | 119/190/75 | 60/101/47 |  | 0.90(0.62-1.31) | 0.588 |  | 0.83(0.55-1.26) | 0.379 |  | 0.90(0.71-1.14) | 0.392 |
|  | rs1063582(G>T) | 236/126/22 | 113/80/15 |  | 0.75(0.53-1.05) | 0.093 |  | 0.78(0.40-1.54) | 0.478 |  | 0.80(0.61-1.05) | 0.103 |
|  | rs2280936(C>G) | 241/125/18 | 139/61/8 |  | 1.20(0.84-1.71) | 0.325 |  | 1.23(0.53-2.88) | 0.634 |  | 1.16(0.86-1.57) | 0.320 |
|  | rs2294133(C>T) | 236/116/32 | 121/79/8 |  | 0.87(0.62-1.23) | 0.436 |  | **2.27(1.03-5.03)** | **0.043** |  | 1.03(0.79-1.36) | 0.822 |
|  | rs2280935(A>C) | 155/177/52 | 82/98/28 |  | 0.96(0.68-1.36) | 0.823 |  | 1.01(0.61-1.65) | 0.978 |  | 0.98(0.77-1.26) | 0.883 |
|  | rs1010156(T>C) | 103/197/84 | 55/104/49 |  | 0.98(0.67-1.44) | 0.920 |  | 0.91(0.61-1.36) | 0.640 |  | 0.96(0.75-1.22) | 0.732 |
|  | rs142252012(G>A) | 373/11/0 | 202/6/0 |  | 0.99(0.36-2.72) | 0.989 |  | - | - |  | 0.99(0.36-2.72) | 0.989 |
|  |  |  |  |  |  |  |  |  |  |  |  |  |
| *LOXL3* | rs715407(T>G) | 252/121/11 | 138/64/6 |  | 1.03(0.72-1.48) | 0.860 |  | 0.99(0.36-2.72) | 0.989 |  | 1.02(0.75-1.40) | 0.880 |
|  | rs6707302(C>T) | 265/109/10 | 145/59/4 |  | 1.03(0.72-1.49) | 0.860 |  | 1.36(0.42-4.40) | 0.604 |  | 1.05(0.76-1.46) | 0.757 |
|  | rs17010021(T>A) | 166/176/42 | 100/83/25 |  | 1.22(0.87-1.71) | 0.258 |  | 0.90(0.53-1.52) | 0.692 |  | 1.09(0.85-1.40) | 0.514 |
|  | rs17010022(C>G) | 174/167/43 | 81/103/24 |  | 0.77(0.55-1.09) | 0.135 |  | 0.97(0.57-1.64) | 0.901 |  | 0.86(0.67-1.11) | 0.242 |
|  |  |  |  |  |  |  |  |  |  |  |  |  |
| *LOXL4* | rs3793692(G>A) | 86/206/92 | 58/105/45 |  | 1.34(0.91-1.97) | 0.138 |  | 1.14(0.76-1.71) | 0.522 |  | 1.18(0.92-1.51) | 0.188 |
|  | rs1983864(G>T) | 126/192/66 | 79/94/35 |  | 1.25(0.88-1.78) | 0.207 |  | 1.03(0.65-1.61) | 0.911 |  | 1.12(0.88-1.43) | 0.357 |
|  | rs7077266(G>T) | 271/104/9 | 146/58/4 |  | 0.98(0.68-1.42) | 0.923 |  | 1.22(0.37-4.02) | 0.739 |  | 1.00(0.72-1.39) | 0.993 |

SNP, single nucleotide polymorphism; OR, odds ratio; CI, confidence interval; −, not available;

^a^Genotype presented as wild type/heterozygous/homozygous; ^b^Control individuals were 208 Chinese Han Population in 1000 Genome Project; Bold font indicates p<0.05.

| **Supplementary Table 3. Univariate logistic regression analysis of associations of polymorphisms in *LOX* family genes and risk of single IA in the additional Chinese population controls** | | | | | | | | | | | | |
| --- | --- | --- | --- | --- | --- | --- | --- | --- | --- | --- | --- | --- |
| GENE | SNP | Genotype^a^ | |  | Dominant model | |  | Recessive model | |  | Additive model | |
|  |  | Case (n) | Control (n)^b^ |  | OR (95%CI) | *P* value |  | OR (95%CI) | *P* value |  | OR (95%CI) | *P* value |
| *LOX* | rs1800449(C>T) | 163/72/13 | 132/64/12 |  | 0.91(0.62-1.33) | 0.614 |  | 0.90(0.40-2.03) | 0.805 |  | 0.92(0.68-1.26) | 0.616 |
|  | rs2956540(G>C) | 137/87/24 | - |  | - | - |  | - | - |  | - | - |
|  | rs10519694(C>T) | 201/32/15 | 184/24/0 |  | **1.79(1.05-3.05)** | **0.031** |  | - | - |  | **1.97(1.26-3.09)** | **0.003** |
|  | rs2303656(G>T) | 233/15/0 | 192/15/1 |  | 0.77(0.37-21.60) | 0.488 |  | - | - |  | 0.74(0.37-1.48) | 0.394 |
|  | rs763497(A>G) | 177/64/7 | 150/50/8 |  | 1.04(0.69-1.56) | 0.860 |  | 0.73(0.26-2.04) | 0.543 |  | 0.99(0.70-1.4) | 0.955 |
|  | rs3900446(A>G) | 203/40/5 | 179/28/1 |  | 1.37(0.82-2.27) | 0.227 |  | 4.26(0.49-36.75) | 0.188 |  | 1.41(0.89-2.24) | 0.142 |
|  |  |  |  |  |  |  |  |  |  |  |  |  |
| *LOXL1* | rs2165241(C>T) | 201/43/4 | 178/29/1 |  | 1.39(0.84-2.29) | 0.200 |  | 3.39(0.38-30.60) | 0.276 |  | 1.41(0.89-2.23) | 0.146 |
|  | rs3825942(G>A) | 186/56/6 | 157/46/5 |  | 1.03(0.67-1.57) | 0.906 |  | 1.01(0.30-3.34) | 0.991 |  | 1.02(0.70-1.48) | 0.915 |
|  | rs2304721(C>A) | 141/84/23 | 115/77/16 |  | 0.94(0.65-1.36) | 0.737 |  | 1.23(0.63-2.39) | 0.548 |  | 1.00(0.75-1.33) | 0.998 |
|  | rs12441130(T>C) | 116/97/35 | 93/85/30 |  | 0.92(0.64-1.33) | 0.660 |  | 0.98(0.58-1.65) | 0.925 |  | 0.95(0.74-1.24) | 0.721 |
|  |  |  |  |  |  |  |  |  |  |  |  |  |
| *LOXL2* | rs2294128(C>T) | 198/54/4 | 142/63/3 |  | **0.54(0.36-0.83)** | **0.005** |  | 1.12(0.25-5.06) | 0.883 |  | **0.61(0.41-0.90)** | **0.012** |
|  | rs7818494(A>G) | 158/74/16 | 127/76/5 |  | 0.89(0.61-1.31) | 0.560 |  | **2.80(1.01-7.78)** | **0.048** |  | 1.04(0.76-1.43) | 0.798 |
|  | rs4323477(A>G) | 60/129/59 | 51/97/60 |  | 1.02(0.66-1.56) | 0.936 |  | 0.77(0.51-1.71) | 0.221 |  | 0.91(0.70-1.18) | 0.479 |
|  | rs7818416(G>A) | 92/110/46 | 60/101/47 |  | 0.69(0.46-1.02) | 0.063 |  | 0.78(0.49-1.23) | 0.286 |  | 0.79(0.61-1.02) | 0.070 |
|  | rs1063582(G>T) | 154/85/9 | 113/80/15 |  | 0.73(0.50-1.05) | 0.094 |  | 0.49(0.21-1.13) | 0.094 |  | **0.73(0.53-0.99)** | **0.043** |
|  | rs2280936(C>G) | 153/85/10 | 139/61/8 |  | 1.25(0.85-1.84) | 0.256 |  | 1.05(0.41-2.71) | 0.919 |  | 1.18(0.85-1.64) | 0.317 |
|  | rs2294133(C>T) | 148/74/26 | 121/79/8 |  | 0.94(0.65-1.37) | 0.745 |  | **2.93(1.30-6.6203)** | **0.010** |  | 1.14(0.85-1.53) | 0.388 |
|  | rs2280935(A>C) | 101/111/36 | 82/98/28 |  | 0.95(0.65-1.38) | 0.777 |  | 1.09(0.64-1.86) | 0.747 |  | 0.99(0.76-1.30) | 0.969 |
|  | rs1010156(T>C) | 75/118/55 | 55/104/49 |  | 0.83(0.55-1.25) | 0.371 |  | 0.93(0.60-1.43) | 0.726 |  | 0.90(0.70-1.17) | 0.441 |
|  | rs142252012(G>A) | 241/7/0 | 202/6/0 |  | 0.98(0.32-2.96) | 0.968 |  | - | - |  | 0.98(0.32-2.96) | 0.968 |
|  |  |  |  |  |  |  |  |  |  |  |  |  |
| *LOXL3* | rs715407(T>G) | 161/79/8 | 138/64/6 |  | 1.07(0.72-1.57) | 0.749 |  | 1.12(0.38-3.29) | 0.833 |  | 1.06(0.76-1.49) | 0.730 |
|  | rs6707302(C>T) | 168/73/7 | 145/59/4 |  | 1.10(0.74-1.63) | 0.652 |  | 1.48(0.43-5.13) | 0.535 |  | 1.11(0.78-1.59) | 0.559 |
|  | rs17010021(T>A) | 112/111/25 | 100/83/25 |  | 1.12(0.77-1.63) | 0.534 |  | 0.82(0.46-1.48) | 0.510 |  | 1.02(0.78-1.35) | 0.877 |
|  | rs17010022(C>G) | 110/112/26 | 81/103/24 |  | 0.80(0.55-1.16) | 0.244 |  | 0.90(0.50-1.62) | 0.720 |  | 0.86(0.65-1.14) | 0.296 |
|  |  |  |  |  |  |  |  |  |  |  |  |  |
| *LOXL4* | rs3793692(G>A) | 53/135/60 | 58/105/45 |  | 1.43(0.93-2.19) | 0.107 |  | 1.16(0.75-1.80) | 0.520 |  | 1.21(0.93-1.59) | 0.161 |
|  | rs1983864(G>T) | 73/127/48 | 79/94/35 |  | 1.47(0.99-2.17) | 0.054 |  | 1.19(0.73-1.92) | 0.486 |  | 1.25(0.96-1.63) | 0.094 |
|  | rs7077266(G>T) | 170/73/5 | 146/58/4 |  | 1.08(0.72-1.61) | 0.705 |  | 1.05(0.28-3.96) | 0.943 |  | 1.07(0.74-1.54) | 0.717 |

SNP, single nucleotide polymorphism; OR, odds ratio; CI, confidence interval; −, not available;

^a^Genotype presented as wild type/heterozygous/homozygous; ^b^Control individuals were 208 Chinese Han Population in 1000 Genome Project; Bold font indicates p<0.05.

| **Supplementary Table 4. Univariate logistic regression analysis of associations of polymorphisms in *LOX* family genes and risk of multiple IA in the additional Chinese population controls** | | | | | | | | | | | | |
| --- | --- | --- | --- | --- | --- | --- | --- | --- | --- | --- | --- | --- |
| GENE | SNP | Genotype^a^ | |  | Dominant model | |  | Recessive model | |  | Additive model | |
|  |  | Case (n) | Control (n)^b^ |  | OR (95%CI) | *P* value |  | OR (95%CI) | *P* value |  | OR (95%CI) | *P* value |
| *LOX* | rs1800449(C>T) | 74/53/9 | 132/64/12 |  | 1.46(0.94-2.26) | 0.095 |  | 1.16(0.47-2.83) | 0.748 |  | 1.30(0.92-1.85) | 0.142 |
|  | rs2956540(G>C) | 58/60/18 | - |  | - | - |  | - | - |  | - | - |
|  | rs10519694(C>T) | 112/18/6 | 184/24/0 |  | 1.64(0.89-3.03) | 0.112 |  | - | - |  | **1.85(1.09-3.15)** | **0.023** |
|  | rs2303656(G>T) | 115/21/0 | 192/15/1 |  | **2.19(1.01-4.37)** | **0.026** |  | - | - |  | **1.98(1.02-3.84)** | **0.045** |
|  | rs763497(A>G) | 96/33/7 | 150/50/8 |  | 1.08(0.67-1.74) | 0.759 |  | 1.36(0.48-3.83) | 0.565 |  | 1.10(0.74-1.61) | 0.644 |
|  | rs3900446(A>G) | 102/32/2 | 179/28/1 |  | **2.06(1.19-3.57)** | **0.010** |  | 3.09(0.28-34.41) | 0.359 |  | **1.98(1.18-3.33)** | **0.010** |
|  |  |  |  |  |  |  |  |  |  |  |  |  |
| *LOXL1* | rs2165241(C>T) | 102/33/1 | 178/29/1 |  | **1.98(1.14-3.42)** | **0.015** |  | 1.53(0.10-24.72) | 0.763 |  | **1.89(1.12-3.19)** | **0.018** |
|  | rs3825942(G>A) | 98/34/4 | 157/46/5 |  | 1.19(0.73-1.95) | 0.479 |  | 1.23(0.32-4.67) | 0.761 |  | 1.17(0.76-1.78) | 0.478 |
|  | rs2304721(C>A) | 82/49/5 | 115/77/16 |  | 0.81(0.53-1.26) | 0.359 |  | 0.46(0.16-1.28) | 0.137 |  | 0.78(0.54-1.12) | 0.181 |
|  | rs12441130(T>C) | 64/57/15 | 93/85/30 |  | 0.91(0.59-1.40) | 0.669 |  | 0.74(0.38-1.43) | 0.363 |  | 0.89(0.65-1.21) | 0.453 |
|  |  |  |  |  |  |  |  |  |  |  |  |  |
| *LOXL2* | rs2294128(C>T) | 100/34/2 | 142/63/3 |  | 0.78(0.48-1.25) | 0.297 |  | 1.02(0.17-6.18) | 0.983 |  | 0.80(0.52-1.26) | 0.337 |
|  | rs7818494(A>G) | 83/47/6 | 127/76/5 |  | 1.00(0.64-1.56) | 0.996 |  | 1.87(0.56-6.27) | 0.308 |  | 1.07(0.72-1.58) | 0.739 |
|  | rs4323477(A>G) | 39/66/31 | 51/97/60 |  | 0.81(0.50-1.32) | 0.391 |  | 0.73(0.44-1.20) | 0.214 |  | 0.82(0.61-1.11) | 0.203 |
|  | rs7818416(G>A) | 51/56/29 | 60/101/47 |  | 0.68(0.43-1.07) | 0.094 |  | 0.93(0.55-1.57) | 0.781 |  | 0.83(0.62-1.12) | 0.218 |
|  | rs1063582(G>T) | 82/41/13 | 113/80/15 |  | 0.78(0.51-1.22) | 0.783 |  | 1.36(0.63-2.96) | 0.438 |  | 0.92(0.65-1.28) | 0.609 |
|  | rs2280936(C>G) | 88/40/8 | 139/61/8 |  | 1.10(0.70-1.73) | 0.685 |  | 1.56(0.57-4.27) | 0.384 |  | 1.13(0.78-1.65) | 0.512 |
|  | rs2294133(C>T) | 88/42/6 | 121/79/8 |  | 0.76(0.49-1.19) | 0.226 |  | 1.15(0.39-3.40) | 0.795 |  | 0.83(0.57-1.22) | 0.345 |
|  | rs2280935(A>C) | 63/57/16 | 82/98/28 |  | 0.75(0.49-1.17) | 0.206 |  | 0.86(0.45-1.65) | 0.645 |  | 0.83(0.60-1.14) | 0.253 |
|  | rs1010156(T>C) | 28/29/79 | 55/104/49 |  | 1.39(0.83-2.33) | 0.216 |  | 0.88(0.52-1.48) | 0.629 |  | 1.08(0.79-1.48) | 0.631 |
|  | rs142252012(G>A) | 132/4 | 202/6/0 |  | 1.02(0.28-3.872) | 0.976 |  | - | - |  | 1.02(0.28-3.68) | 0.976 |
|  |  |  |  |  |  |  |  |  |  |  |  |  |
| *LOXL3* | rs715407(T>G) | 91/42/3 | 138/64/6 |  | 0.98(0.62-1.54) | 0.913 |  | 0.76(0.19-3.09) | 0.701 |  | 0.96(0.64-1.44) | 0.832 |
|  | rs6707302(C>T) | 97/36/3 | 145/59/4 |  | 0.93(0.58-1.49) | 0.749 |  | 1.15(0.25-5.22) | 0.856 |  | 0.95(0.62-1.46) | 0.812 |
|  | rs17010021(T>A) | 55/64/17 | 100/83/25 |  | 1.36(0.88-2.11) | 0.165 |  | 1.05(0.54-2.02) | 0.894 |  | 1.19(0.87-1.63) | 0.281 |
|  | rs17010022(C>G) | 64/55/17 | 81/103/24 |  | 0.72(0.46-1.11) | 0.137 |  | 1.10(0.57-2.13) | 0.788 |  | 0.85(0.62-1.18) | 0.334 |
|  |  |  |  |  |  |  |  |  |  |  |  |  |
| *LOXL4* | rs3793692(G>A) | 36/68/32 | 58/105/45 |  | 1.07(0.66-1.75) | 0.774 |  | 1.12(0.67-1.87) | 0.680 |  | 1.07(0.79-1.45) | 0.670 |
|  | rs1983864(G>T) | 54/64/18 | 79/94/35 |  | 0.93(0.60-1.45) | 0.748 |  | 0.75(0.61-1.39) | 0.368 |  | 0.90(0.66-1.22) | 0.490 |
|  | rs7077266(G>T) | 101/31/4 | 146/58/4 |  | 0.82(0.50-1.33) | 0.412 |  | 1.55(0.38-6.29) | 0.543 |  | 0.89(0.58-1.36) | 0.586 |

SNP, single nucleotide polymorphism; OR, odds ratio; CI, confidence interval; −, not available;

^a^Genotype presented as wild type/heterozygous/homozygous; ^b^Control individuals were 208 Chinese Han Population in 1000 Genome Project; Bold font indicates p< 0.05.
